# Nestin Reporter Transgene Labels Multiple Central Nervous System Precursor Cells

**DOI:** 10.1155/2010/894374

**Published:** 2011-03-09

**Authors:** Avery S. Walker, Gwendolyn E. Goings, Yongsoo Kim, Richard J. Miller, Anjen Chenn, Francis G. Szele

**Affiliations:** ^1^Department of Pediatrics, Feinberg School of Medicine, Northwestern University, Chicago, IL 60611-3008, USA; ^2^Department of Physiology, Anatomy and Genetics, University of Oxford, Oxford, UK; ^3^Cold Spring Harbor Laboratory, One Bungtown Road, Cold Spring Harbor, NY 11724, USA; ^4^Department of Molecular Pharmacology and Biological Chemistry, Feinberg School of Medicine, Northwestern University, Chicago, IL 60611-3008, USA; ^5^Department of Pathology, Feinberg School of Medicine, Northwestern University, Chicago, IL 60611-3008, USA

## Abstract

Embryonic neuroepithelia and adult subventricular zone (SVZ) stem and progenitor cells express nestin. We characterized a transgenic line that expresses enhanced green fluorescent protein (eGFP) specified to neural tissue by the second intronic enhancer of the nestin promoter that had several novel features. During embryogenesis, the dorsal telencephalon contained many and the ventral telencephalon few eGFP+ cells. eGFP+ cells were found in postnatal and adult neurogenic regions. eGFP+ cells in the SVZ expressed multiple phenotype markers, glial fibrillary acidic protein, Dlx, and neuroblast-specific molecules suggesting the transgene is expressed through the lineage. eGFP+ cell numbers increased in the SVZ after cortical injury, suggesting this line will be useful in probing postinjury neurogenesis. In non-neurogenic regions, eGFP was strongly expressed in oligodendrocyte progenitors, but not in astrocytes, even when they were reactive. This eGFP+ mouse will facilitate studies of proliferative neuroepithelia and adult neurogenesis, as well as of parenchymal oligodendrocytes.

## 1. Introduction

Expression of the intermediate filament protein nestin has been used as a marker for neural stem and progenitor cells in the ventricular and subventricular zones [1–3]. Nestin is also expressed by radial glia [1] which are substrates for migration and which can give rise to neurons [4, 5]. The second intronic enhancer of nestin specifies expression of the gene to neural tissues [6]. We generated a transgenic mouse using the second intronic enhancer of the nestin gene and the thymidine kinase minimal promoter to drive enhanced green fluorescent protein (eGFP+), an approach similar to what has been successfully used by others (Table 1) [7–12]. Several useful nestin-Cre mice have also been made allowing lineage studies and functional studies [13–15]; however for space constraints, we did not include them in Table 1. 

Transgenic reporter mice, even when generated identically, can have widely divergent expression of the transgene [16]. Indeed, we discovered some aspects of eGFP expression that differed from previously reported lines. In our transgenic mouse, eGFP was expressed more robustly in the pallium than the subpallium during embryogenesis. In the adult SVZ, eGFP+ cells expressed markers of multiple cell subtypes. Interestingly, in the ventral lateral ventricle, eGFP was primarily expressed by ependymal cells. Unexpectedly, eGFP was also detected in oligodendrocytes in the parenchyma throughout development and in the adult. However, it was not expressed in astrocytes, even after cortical injuries rendered them reactive. Thus, our novel eGFP+ mouse will be useful for studies of neurogenesis as well as oligodendrocyte genesis.

## 2. Results

### 2.1. Embryonic Expression of eGFP in Proliferative Neuroepithelia

**Figure 1:**
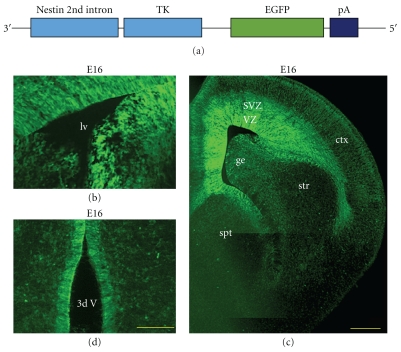
Embryonic proliferative neuroepithelia express eGFP. (a) Transgenic construct. (b) eGFP is expressed at high levels in the VZ and SVZ at E16. (c) Photomontage of coronal hemisection shows typical bright labelling of proliferative neuroepithelium in the developing cortex. Scale bar = 250 microns. (d) eGFP labels VZ cells in the third ventricle. Scale bar = 100 microns.

Nestin, an intermediate filament protein, is expressed by neural stem and progenitor cells [3, 9, 17, 18]. We generated a transgenic mouse that has eGFP [19] specified to neural tissue by the nestin second intronic enhancer and is driven by the minimal thymidine kinase promoter (Figure 1(a)) [6]. During embryogenesis, eGFP was found in expected regions: the large majority of embryonic ventricular zone and subventricular zone cells were labeled (Figure 1(b)). The upper layers of the developing cortex contained eGFP+ processes as well as somata. Unexpectedly, the transgene was driven much less robustly in the proliferative neuroepithelium of the striatum (subpallium) than of the cerebral cortex (pallium) (Figure 1(c)). GFP+ cells were also found in the ventricular zone of more caudal regions such as the third ventricle (Figure 1(d)).

### 2.2. Postnatal Expression of eGFP in Neurogenic Regions

**Figure 2:**
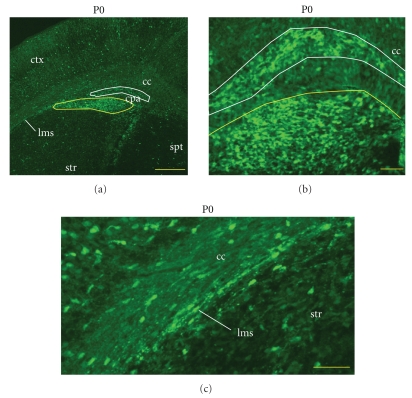
eGFP is expressed in the caudatopallial angle and in the lateral migratory stream at P0. (a) Bright eGFP expression in the caudatopallial angle (cpa). Overall GFP labelling at P0 has diminished. Scale bar = 250 microns. (b) Two discontinuous populations of bright eGFP+ cells are found in the CPA (outlined in yellow and white in A, B). Scale bar = 50 microns. (c) The lateral migratory stream (lms) between the corpus callosum and striatum. Scale bar = 50 microns.

**Figure 3:**
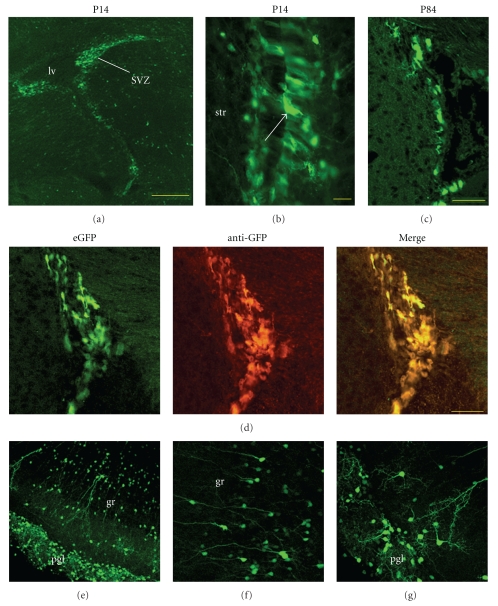
Postnatal and adult neurogenic regions express eGFP. (a) The P14 SVZ is replete with eGFP+ cells. Scale bar = 250 microns. (b) The majority of eGFP+ cells surounding the ventral lateral ventricle are in the ependymal layer (arrow). Scale bar = 20 microns. (c) eGFP expression is still robust in the SVZ at P84. Scale bar = 100 microns. (d) Excellent overlap between eGFP fluoresecence and GFP detected with antibodies (P84 SVZ). Scale bar = 50 microns. (e)–(g) Olfactory bulb labeling. eGFP transgene labels newborn neurons in the granule (gr) (f) and periglomerular (pgl) (g) layers.

**Figure 4:**
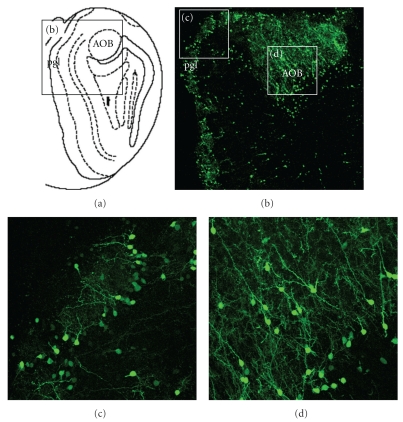
eGFP is expressed in the accessory olfactory bulb. (a) Schematic showing location of accessory olfactory bulb (AOB). Note that at this level (caudal OB) the periglomerular layer is only found medially. Boxed area shows location of (b). (b) Low magnification showing numerous eGFP+ cells in the periglomerular layer and AOB. Boxed areas show locations of (c) and (d). (c), (d) Higher magnification showing morphology of neurons. P42 mouse.

At P0, many eGFP+ cells were found in the caudatopallial angle and lateral migratory stream (Figure 2). These cells seemed to be distributed in two distinct clusters (Figure 2(a)). At P14, many eGFP+ cells were found in the dorsal SVZ (Figure 3(a)), in the rostral migratory stream, and in the subgranular zone of the dentate gyrus (not shown). Unexpectedly, eGFP+ cells surrounding the ventral lateral ventricles were primarily in the ependymal layer (Figure 3(b)). eGFP+ expression was retained in the adult SVZ although relatively fewer cells were labeled (Figure 3(c)). eGFP+ cells in the dorsal SVZ and RMS had a migratory morphology (Figure 3(d)): oval cell bodies with a long leading process. eGFP+ cells with migratory, or adult neuronal morphologies were also found in all layers of the OB (Figures 3(e)–3(g)) and accessory olfactory bulb (Figure 4). These results suggested that eGFP labeled migratory and differentiating SVZ-derived neuroblasts.

Another report of nestin-eGFP mice suggested that eGFP fluorescence intensity was either dim or bright [12]. In our mice, eGFP fluorescence ranged in a continuum from barely visible to quite bright (ex. Figure 3(d)). In order to confirm that we were detecting the transgene and not exciting anomalous (e.g., paraformaldehyde-induced) autofluorescence, we immunolabeled with antibodies against GFP. The anti-GFP immunolabel matched the eGFP signal (Figure 3(d)).

### 2.3. Phenotypic Characterization of eGFP Cells in the Postnatal SVZ

**Figure 5:**
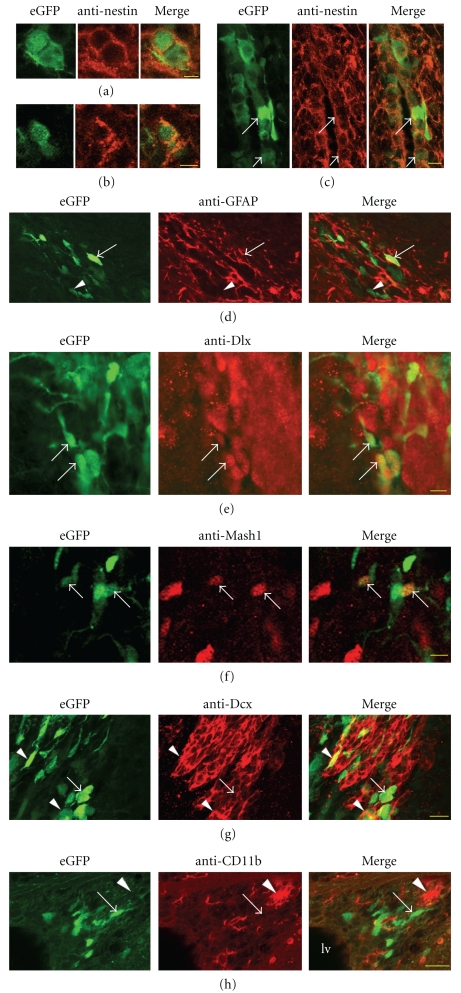
Phenotypic identification of eGFP+ cells in the adult SVZ. (a) eGFP+ cells immunolabeled with antinestin antibody; SVZ, P84. Cells appear to have just gone through cytokinesis. Scale bar = 10 microns. (b) eGFP+ cell immunolabeled with antinestin antibody; SVZ, P84. Scale bar = 5 microns. (c) Long arrow shows bright, and short arrow dim, eGFP+ cells in the ventral ependymal layer of the lateral ventricle labeled with antinestin antibody. Scale bar = 10 microns. (d) eGFP+ cell (arrow) expressing GFAP immunoreactivity in the SVZ of a P28 mouse. Bright cell indicated with arrow has nucleus filled with eGFP and is surrounded by GFAP in extending processes. Other cells (arrowhead) are in GFAP-negative areas. (e) Some eGFP+ cells coexpress the transcription factor Dlx. Scale bar = 10 microns. (f) eGFP+ could also be Mash1+. Scale bar = 10 microns. (g) Some eGFP+ cells (arrowheads) coexpress doublecortin in the RMS, whereas others (arrow) do not. Scale bar = 50 microns. (h) eGFP+ cells in the SVZ (arrow) do not express the microglial marker CD11b+ microglia (arrowhead). Scale bar = 50 microns.

**Figure 6:**
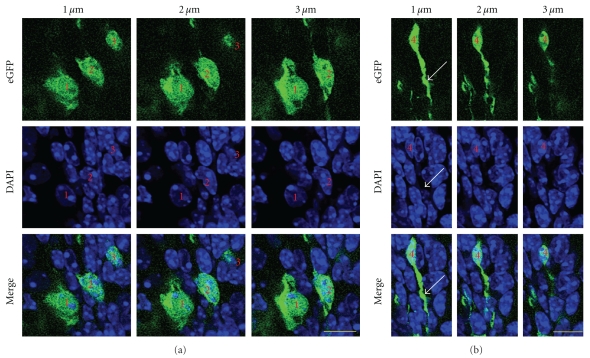
Nuclear and cytoplasmic localization of eGFP transgene. (a) and (b) Series of confocal optical sections spaced 1 micron apart, from P84 SVZ. Four cells are shown. eGFP is found in the nucleus, perinuclear cytoplasm and in processes extending away from the nucleus. Scale bars = 20 microns.

Endogenous nestin has been reported in all three of the major neural SVZ cells (stem cells, progenitors, and neuroblasts) [20]. We first sought to characterize the transgene expression by comparing eGFP fluorescence with nestin immunohistochemistry. Nestin immunolabeling revealed the cytoplasm and processes of cells in the SVZ and ependymal layer (Figures 5(a)–5(c)). Conversely, eGFP was largely found in the cell body and short processes (Figures 6(a)–6(b)). For unknown reasons, low levels of eGFP were also detected with confocal microscopy in the nucleus of SVZ cells (Figure 6). Close examination showed many eGFP+ cells that were also labeled by the antinestin antibody (Figures 5(a)–5(c)). This was similar to other nestin reporter mice and was therefore expected. We next tested whether eGFP was coexpressed in the GFAP-expressing cells of the adult SVZ [21]. GFAP immunolabelling in the SVZ was fibrous and tangled, similar to nestin (Figure 5(d)). This made eGFP/GFAP colocalization difficult; however, we did find rare eGFP+ cells that were GFAP positive (Figure 5(d)). We hypothesized that some eGFP+ cells in the SVZ would be type C progenitor cells [20, 21]. We found eGFP+ cells in the SVZ that expressed epidermal growth factor receptor, a type C cell marker (not shown). The transcription factors Dlx and Mash1 are mostly expressed by SVZ type C progenitors [22, 23], and we found double-labeled cells (Figures 5(e), 5(f)). We also found many eGFP+ cells that expressed doublecortin (Dcx), a marker of migrating neuroblasts (Figure 5(f)). A few reports have indicated that nestin may be expressed by microglial cells [24–26]. We examined whether eGFP+ cells in the SVZ express the microglial markers CD11b or CD45. Microglia frequently contacted eGFP+ cells; however we did not find any double-labeled cells in the SVZ (Figure 5(g).

**Figure 7:**
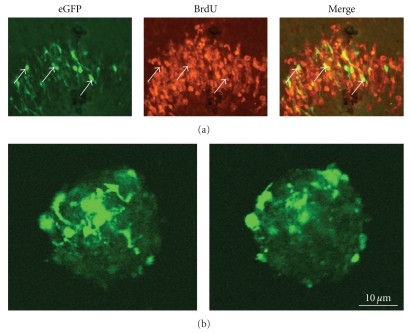
eGFP+ cells are proliferative and generate neurospheres. (a) The majority of eGFP+ cells in the RMS are positive for BrdU. BrdU was administered for two weeks daily, and mice sacrificed the last day of BrdU. (b) eGFP+ cells in neurospheres prepared from eGFP+ mice.

We next studied the mitotic potential of eGFP+ cells by administering BrdU for two weeks in four-week-old mice. eGFP+ cells in the postnatal SVZ and RMS (Figure 7(a)) were labeled with BrdU, indicating that they had been born during that period. Interestingly, only a small fraction of BrdU+ cells were eGFP+, suggesting that this transgene only labels a subset of newly generated cells in the postnatal SVZ. Neurospheres generated *in vitro* from proliferative neuroepithelia can exhibit self-renewal and multipotency, the two cardinal features of stem cells. We examined the ability of eGFP+ cells to make neurospheres. Similar to other nestin-GFP lines [8, 9, 12], the SVZ from these eGFP+ mice generated many green neurospheres (Figure 7(b)) that could be passaged multiple times. Since we also found neurospheres that did not express eGFP, it is likely that some of the neurospheres formed from eGFP-negative SVZ stem cells. This was expected since in this line of mice, eGFP is not found in all SVZ precursor cells. These neurosphere data are compatible with our phenotypic analysis and suggest that a portion of eGFP+ cells in the SVZ are stem cells.

### 2.4. Oligodendrocyte Precursors Express eGFP

**Figure 8:**
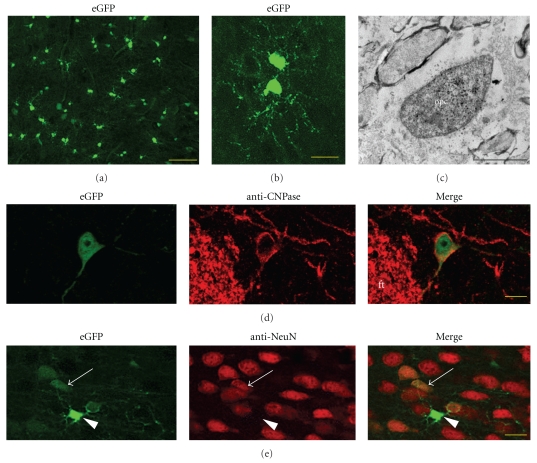
Oligondendrocyte progenitors express eGFP. (a) P14 piriform cortex contains multiple bright eGFP+ cells with the morphology of oligodendrocyte progenitor cells (OPC). Note the numerous pairs of cells. Scale bar = 50 microns. (b) Two eGFP+ cells showing typical morphology of OPC's. (P28 mouse, near base of lateral ventricles). Scale bar = 20 microns. (c) eGFP+ immunoreactive cell with the ultrastructural features of an oligodendrocyte. Scale bar = 2 microns. (d) eGFP+ striatal cell that is immunolabeled with the oligodendrocyte marker CNPase (P28 mouse). CNPase antibodies also labeled white matter tracts like the corticostriate fiber tract (ft) seen in this panel. Scale bar = 10 microns. (e) Oligodendrocyte-like eGFP+ cell (arrowhead) does not express NeuN. This cell wraps processes around an adjacent NeuN+ neuron. Note that a few NeuN+ neurons (arrow) exhibit weak eGFP fluorescence. Scale bar = 20 microns.

eGFP was also expressed by scattered cells throughout the brain parenchyma at all ages examined, and were especially dense in the piriform cortex (Figure 8(a)). Many of these cells were doublets suggesting that they had recently divided (Figure 8(b)). Immunoelectron microscopy with anti-GFP antibodies showed these cells had ultrastructural features of oligodendrocytes (Figure 8(c)). Previous work from our laboratory had used EM to delineate SVZ cells that expressed eGFP. We also found some eGFP+ cells in the parenchyma that expressed the oligodendrocyte marker CNPase and which were found as individual cells (Figure 8(d)). Most of the CNPase+/eGFP+ cells expressed the transgene at rather low levels. Other eGFP+ cells scattered throughout the brain were dim and expressed the neuronal marker NeuN (Figure 8(e)). The eGFP variant we used is particularly resistant to degradation [19]; therefore, the dim eGFP in neurons may have retained eGFP. Immunolabelling with anti-GFP antibodies confirmed the weak neuronal retention of the transgene (data not shown). We also found bright eGFP+ cells with processes enveloping NeuN+ cells (Figure 8(e)).

### 2.5. Reactive Astrocytes Do Not Express eGFP

**Figure 9:**
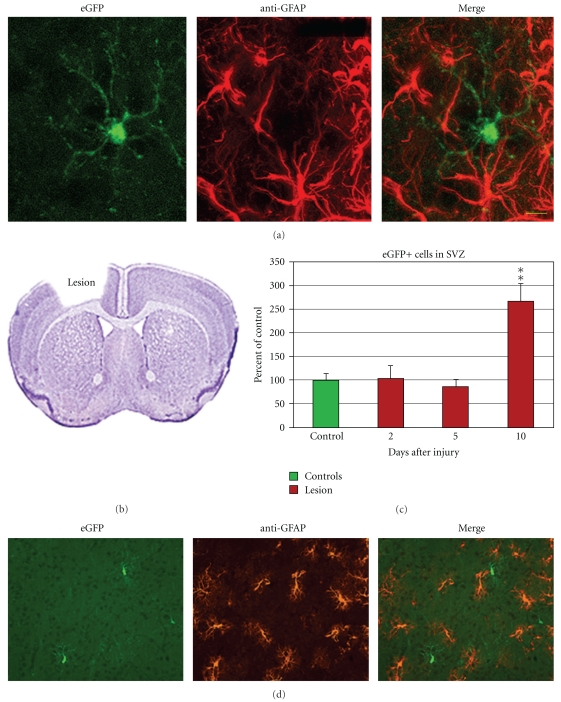
Parenchymal astrocytes do not express eGFP. (a) GFAP+ astrocytes are not constitutively labeled with eGFP transgene (P84 striatum). Scale bar = 20 microns. (b) Cortical lesions (les) were performed to determine if reactive astrocytes express eGFP. Coronal section adapted from Paxinos' atlas (Paxinos and Franklin, 2001). (c) The number of eGFP+ cells in the SVZ increased 10 days after injury (**P < .01, *t*-Test). (d) eGFP+ cells in the denervated striatum did not colabel with GFAP immunohistochemistry (reactive astrocytes 10 days after injury).

eGFP was expressed in oligodendrocyte progenitors; in addition, some eGFP+ cells in the SVZ were GFAP+. Therefore, we asked whether eGFP was expressed in astrocytes outside of the SVZ, in the parenchyma. We labeled with anti-GFAP antibodies and did not find any double labeled cells (Figure 9(a)).

Reactive astrocytes have been reported to express nestin after certain lesion paradigms [24, 27]. To address whether eGFP+ astrocytes appear after injury, we performed cortical aspiration lesions (Figure 9(b)). Interestingly, the number of eGFP+ cells increased in the SVZ at 10 days after injury (Figure 9(c)). We next performed GFAP immunohistochemistry to detect reactive astrocytes. As previously shown [28], astrocytosis occurred around the lesion, in the corpus callosum, and in the striatum (Figure 9(d)). In contrast, we saw no evidence of reactive astrocytosis in the SVZ proper (though the lesions were close to the SVZ, they did not extend into it). We quantified eGFP+ cells in the striatum, corpus callosum, and cerebral cortex in controls (*N* = 4 mice, 89 cells examined), at 2 days (*N* = 3, 74 cells), 5 days (*N* = 4, 100 cells), and 10 days (*N* = 4, 104 cells) after injury. We did not find a single eGFP+ cell in these regions that was colabeled with GFAP (Figure 9(d)). Thus, whereas eGFP is expressed by some GFAP+ cells in the SVZ, eGFP is not expressed by astrocytes outside of the SVZ even when they become reactive after injury. Therefore, use of this mouse to examine neurogenesis will not be confounded by the expression of eGFP in reactive astrocytes.

## 3. Discussion

We have characterized a transgenic mouse in which the expression of enhanced green fluorescent protein is specified by the second intronic enhancer of the nestin gene and driven by the thymidine kinase minimal promoter. eGFP was found in developmental and adult proliferative neuroepithelia; the regions within which we expected to find it based on endogenous nestin expression and other nestin reporter mice [1, 8, 9, 12]. In the following respects eGFP expression in this line differed (Table 1) from previously described reporter lines: it exhibited dorsoventral heterogeneity in neurogenic regions, and it was expressed by newborn OB neurons and oligodendrocytes, but was not expressed by astrocytes or microglia.

eGFP was expressed at higher levels during embryogenesis in pallial compared to subpallial proliferative neuroepithelia. It may be that other nestin reporter mice also exhibited dorsoventral differences in eGFP expression, but were merely not reported. We also found that the dorsal lateral ganglionic eminence preferentially expressed eGFP at high levels. This lens-shaped cluster of eGFP+ cells is in the same location as the Er81+ intermediate domain that gives rise to a subset of adult SVZ cells [29]. This eGFP+ domain is contiguous with the lateral migratory stream, which extends from the intermediate domain towards the basolateral forebrain [30]. Dorsoventral differences were also found in the adult: dorsal eGFP was primarily expressed in the SVZ, whereas ventrally, it was primarily expressed in ependymal cells.

We showed previously that the ventral lateral ventricle in adult mice is characterized by cells in the SVZ and ependymal layer that extend long nestin, vimentin, and GFAP-positive radial glia-like processes into the surrounding neuropil [31]. Interestingly, Dcx+ cells appear to migrate from the ventral SVZ population amongst the radial glia-like fibers into the adjacent neuropil [31, 32]. It has been proposed that ependymal cells lining the lateral ventricles are also stem cells in this system [33]. Although most labs have not been able to confirm that ependymal cells normally exhibit stem cell characteristics [34, 35], the possibility exists that the identity of the stem cell phenotype varies in subregions of the ventricular walls. Could it be that the ventral eGFP+ ependyma maintain more stem cell characteristics than dorsally located ependyma? This transgenic mouse line should help solve some of these questions. 

Some nestin+ cells *in vitro* exhibit the stem cell characteristics of self-renewal and multipotency [9, 12]. They are mitotically active *in vivo *and are found at the same locations and times as brain stem cells [8]. The BrdU and neurosphere studies we performed showed that many of the eGFP+ cells were proliferative. In the adult SVZ, a subset of the GFAP+ astrocyte-like cells are thought to be the stem cells [21]. We found eGFP+ cells that expressed GFAP, both *in vivo* and *in vitro*. We used electron microscopy to quantify the percent of SVZ cells expressing eGFP in a previous study [36]. We found that 8% of the eGFP+ cells in the SVZ were astrocyte-like cells, 18% were ependymal cells, 8% were transit amplifying type C cells, and 63% were neuroblasts. It is not clear if these “downstream” cells in the SVZ lineage merely inherited and retained this particularly bright variant of eGFP [19], or if the transgene was actively driven. Either way, in previous studies we showed that the eGFP+ neuroblasts are useful for studying migration [36, 37]. In contrast to [12], we did not notice any correlation between eGFP+ brightness and progression from precursor cells in the SVZ to differentiated cell in the OB. Mignone and colleagues showed that SVZ stem/progenitor cells were GFP-bright whereas neuroblasts were dim. In our line SVZ progenitor cells, and migratory neuroblasts as well as OB neurons exhibited bright eGFP+. The most parsimonious explanation for this discrepancy is that we used a particularly stable variant of eGFP [19]. In the current study it was of particular interest that both periglomerluar and granule neurons, the final product of the SVZ system were eGFP+. This is in contrast with reports by other groups, in which only periglomerular neurons were labeled [11, 12]. Periglomerular cells are a small subset of cells generated by the SVZ; therefore, it is curious that the reporter molecules in the previous reports were only expressed in these neurons [11, 12]. Although we do not know for certain, it is likely that eGFP labeling of granule and periglomerular neurons is due to retention of the eGFP, since nestin mRNA is not detected in these cells. Nestin as a cytoskeletal protein may have a different half-life than GFP. We do not advocate use of this line as a nestin lineage tracer but point out that since it labels multiple OB neurons, it could be useful for their study. Taken together, the data suggest that the mice described here will be useful for visualizing all types of SVZ cells and multiple OB neurons. However, in these mice eGFP is not a specific marker of stem cells but is also found in postmitoitc cells and tissues.

A low level of oligodendrocyte genesis occurs homeostatically in the adult. We found eGFP+ cells scattered in the neural parenchyma that were morphologically similar to oligodendrocytes. They were often found in pairs, which may have been due to recent divisions. Some of these eGFP+ cells expressed CNPase, an oligodendrocyte marker. This is consistent with others finding that another oligodendrocyte marker NG2, [38] and PDGFR*α* were expressed by nestin+ cells [39]. Since the eGFP fluorescence was bright in these parenchymal cells, they are suitable for live imaging studies of oligodendrocyte dynamics.

In addition to oligodendrocytes, astrocytes and microglia may have expressed eGFP [24–27]. However, we found that neither cell type expressed eGFP. Reactive astrocytes are notorious for “aberrantly” expressing nonastrocytic molecules. However, we found that after cortical aspiration lesions, eGFP was not expressed in astrocytes, similar to what others have found [40]. Interestingly these lesions did result in larger numbers of eGFP+ cells in the SVZ, suggesting that the line can be used to monitor SVZ plasticity in response to injury, but that astrocyte expression of the transgene will not be a confounding factor. 

One possible explanation for the differences in the various nestin-reporter mice is background strain differences. For example C57BL/6 mice were used in the study Mignone et al. [12], whereas we used FVB/N's. Another difference is that we relied on the nestin second intronic enhancer followed by the thymidine kinase minimal promoter to drive eGFP, whereas Mignone and colleagues engineered GFP in between the nestin promoter and the nestin second intronic enhancer. In fact, there are several important differences between our nestin reporter mice and others arguing that nestin promoter/enhancer driven transgenes may be vulnerable to positional effects. Although outside the scope of this study careful comparison of integration sites may be revelatory in the future. 

Although this eGFP+ mouse exhibits substantial similarities to other nestin reporter animals, it also exhibits several differences that may be informative biologically and useful technically. The expression in adult ependymal cells suggests that some ependymal cells may indeed be stem cells. Expression of eGFP+ cells in postmitotic brain parenchyma will allow further investigation of these cells.

## 4. Experimental Procedures

### 4.1. eGFP Transgenic Mice

Standard molecular biology techniques were used to generate the transgenic constructs. Briefly, the second intron from the rat nestin gene [6, 41] was placed upstream of the thymidine kinase minimal promoter to drive a modifed form of enhanced green fluorescent protein (EGFPmut4) [19]. DNA was generously provided by the following individuals: nestin/tk, J. M. Hebert, EGFPmut4, A. Okada. Transgenic mice in the FVB/N strain were generated by pronuclear injection, in the transgenic mouse cores at Children's Hospital Boston and Brigham and Women's Hospital. Animals were genotyped by PCR of tail DNA using primers specific to transgene sequences. Two mm of tail was placed into a 1.5 mL microfuge tube with 200 *μ*L 1x PBND buffer (Jackson Labs) and 1 *μ*L of 10 mg/mL Proteinase K and incubated at 55°C, overnight, and 1-2*μ*L of the processed, lysed tail DNA was used for each PCR reaction.

### 4.2. Cortical Lesions

Were performed as described previously [42–44]. Adult mice were anesthetized with a mixture of ketamine (100 mg/kg) and xylazine (10 mg/kg) IP, placed in a Cunningham stereotax mouse adapter (Stoelting). The left frontoparietal cerebral cortex from Bregma to 1.2 mm anterior to Bregma was gently aspirated with a fire-polished glass Pasteur pipette attached to a vacuum, to the level of the corpus callosum. Gelfoam was placed in the lesion before suturing. Nonlesioned control mice were anesthetized with ketamine/xylaxine and housed with lesioned animals.

### 4.3. BrdU Administration

Brdu (200 mg/kg) was injected intraperitoneally in 4-week-old eGFP+ mice every 12 hrs for two weeks.

### 4.4. Tissue Preparation

Male and female eGFP+ mice ranging between ages E16-P224 were used: E16 *N* = 4, P0 *N* = 3, P14 *N* = 4, P28 *N* = 1, P30 *N* = 3, P63 *N* = 1, P84 *N* = 4, and P224 *N* = 1. Mice were anesthetized at 4°C with wet ice between E162-P14. Ages P28–P224 were anesthetized with sodium pentobarbital and perfused with 4% paraformaldehyde in 0.1 M sodium phosphate buffered saline (PBS), pH 7.6. Thirty micron free-floating coronal sections were cut on a sliding microtome and stored in cryoprotectant at −20°C. All animal procedures were in accordance with NIH animal care guidelines and were approved by the Children's Memorial Hospital IACUC committee.

### 4.5. Neurosphere Production

The lateral wall of the lateral ventricle, including the SVZ, was dissected from coronal slices of P5 mouse brains, dissociated with 200 mL Papain (4 U/mL; Worthington), and plated at a density of 3,000 cells/mL in 25 cm^2^ flasks coated with Poly-HEME (2.4 mg/cm^2^; Sigma) in Neurobasal (Invitrogen) supplemented with the growth factors fibroblast growth factor (FGF-2) (20 ng/mL; Sigma) and epidermal growth factor (EGF) (20 ng/mL; R&D Systems), as well as B-27 Supplement (Gibco), N-2 Supplement (Gibco), L-glutamine (2 mM; Invitrogen), and Penicillin/Streptomycin (100 U/mL; Gibco). Neurospheres were dissociated and passaged 9 days after seeding. Cultures were kept for at least 4 passages.

### 4.6. Immunohistochemistry

The following antibodies were used: anti-2′,3′-cyclic nucleotide 3′-phosphodiesterase (myelin CNPase; mouse monoclonal, 1 : 1000, Sternberger Monoclonals Inc., Lutherville, MD), antipan Dlx [45] (rabbit polyclonal, 1 : 50, a gift from Dr. J. D. Kohtz, Northwestern Univ.), antidoublecortin (Dcx, goat polyclonal, 1 : 100; Santa Cruz Biotech, Santa Cruz, CA), antiepidermal growth factor receptor (EGFr, mouse monoclonal, 1 : 50; Sigma, St. Louis, MO), antiglial fibrillary acidic protein (GFAP, rabbit polyclonal, 1 : 250; DAKO, Carpinteria, CA), antinestin (mouse monoclonal, 1 : 100; Chemicon, Temecula, CA), antineuronal nuclei (NeuN, mouse monoclonal, 1 : 1000; Chemicon, Temecula, CA), anti-CD45 (rat monoclonal, 1 : 500; Chemicon, Temecula, CA), antibromodeoxyuridine (BrdU, Sheep polyclonal, 1 : 500, Becton Dickinson, San Jose, CA), and antigreen fluorescent protein (GFP, rabbit polyclonal, 1 : 500; Gibco, Calsbad, CA). Sections were blocked with 50 mM glycine in phosphate buffered saline (PBS) to reduce autofluorescence of paraformaldehyde-fixed tissue. Sections were washed, blocked in PBS containing 0–0.1% Triton X-100 and 10% Donkey Serum, DS (Sigma, St. Louis, MO), incubated overnight at 4°C in primary antibody, washed and exposed to 1 : 400 Cy3-conjugate secondary antibodies (Molecular Probes, Eugene, OR) for one hr at RT, washed twice, rinsed in phosphate buffer (PB), mounted on slides, and coverslipped. For BrdU immunohistochemistry, sections were washed in DNAse buffer with 0.5% Triton X-100 for 20 minutes and then incubted w/DNAse 2 mg/mL with 0.5% Trition X-100.

Nuclei were stained by applying 40 mg/mL 4′,6-Diamidino-2-phenylindole dihydrochloride (hydrate) (DAPI, Sigma, St. Louis, MO) for 10 min before the final rinses. Negative controls omitting the primary antibodies were carried out in all experiments for all the secondary antibodies used. Sections were analyzed with Leica epifluorescent microscopes controled by Openlab software (Improvision, Cambridge MA) or an Olympus Confocal microscope controlled by Fluoview software (Olympus, Melville, NY). All GFP+ cells were imaged with 40x or 63x objectives and by shifting between the GFP, Cy3, and DAPI filter cubes. Images generated with these systems were assembled in Adobe Photoshop.

### 4.7. Electron Microscopy

Mice were perfused with 2% paraformaldehyde and 0.1% glutaraldehyde in PBS and left in same fixative overnight at 4°C (*N* = 1 mouse, P45). The brain was vibratome sectioned at 50 *μ*m and washed in PBS, 6x, 10 min. Aldehydes were blocked by 30 min incubation in 50 mM glycine in PBS. Sections were washed in PBS 3 × 10 min, incubated in 5% normal goat serum, 0.1% triton-x 100 in PBS (PBS+), and rabbit anti-GFP 1 : 1000 added for overnight incubation at 4°C. Sections were washed in PBS, 3x, 10 min, incubated for 60 min, RT, in antirabbit conjugated 1.4 nm gold (Nanoprobes, Yaphank, NY) in PBS+. Sections were washed in PBS, 3x, 10 min, PB, 3x, 10 min, DH_2_O, 3x, 10 min, then incubated in Gold Enhance solution (Nanoprobes) for 5 min, washed in DH_2_O, 3x, 5 min, PB 3x, 5 min, fixed in 1% gluteraldehyde for 30 min, washed in PBS, fixed in 1% osmium tetroxide in PBS for 10 min, washed in PBS, dehydrated in EtOH, infiltrated in 1 : 1 propylene oxide : epon for 1 hr at RT, 1 : 2 PO : epon for 2 hrs, epon for 2 hrs, and embedded in epon overnight at 60°C. Stained ultrathin sections in 2% aqueous uranyl acetate for 10 min, washed in DH_2_O, and viewed on a JEOL electron microscope equipped with a digital camera.

## Figures and Tables

**Table 1:** Comparison of nestin-reporter mice.

		Yamaguchi et al. [8]	Aoki et al. [7]	Kawaguchi et al. [9]	Mignone et al. [12]	Beech et al. [11]	Our work
Transgenic strategy	Sequence that drives transgene	2nd Intron, nestin promoter	2nd Intron, thymidine kinase minimal promoter (a)	2nd Intron, hsp68 min promoter	2nd Intron, 5.8 kb nestin promoter	5.8 kb upstream to 5.4 kb downstream of nestin Gene (b)	2nd Intron, thymidine kinase promoter
	Reporter molecule	eGFP	*β*-galactosidase	eGFP	eGFP	CREB	Modified (stable) eGFP (c)

General features	Endogenous nestin coexpression	n.d	Yes	Yes (d)	Yes	n.d. (e)	Yes
	Brdu+	Yes (f)	n.d. (g)	Yes (h)	Yes	Yes	Yes

Embryonic expression	Ventricular zone	Yes	Yes	Yes	Yes	n.d.	Yes
	Subventricular zone	Yes	n.d.	Yes	Yes	n.d.	Yes
	Pallium versus subpallium	n.d.	n.d.	n.d.	No	n.d.	Yes
	3d ventricle	Yes	Yes	Yes	Yes	n.d.	Yes
	Spinal cord	Yes	Yes	Yes	n.d.	n.d.	n.d.
	Postmitotic parencyma	Yes	n.d.	? (i)	No	n.d.	Yes
	Nonneural tissues	No	No	n.d.	No	n.d.	Yes

Postnasal and/or adult expression light microscopy	*Neurogenic regions*	Yes	Yes	Yes	Yes	Yes-subset (j)	Yes
	Dentate gyrus progenitors	Yes	?	n.d.	Yes	No	Yes
	Dentate gyrus neurons	Yes	?	n.d.	No	No	No
	SVZ B cells	Yes	No	Yes (k)	Yes	? (l)	Yes-subset
	SVZ C cells	n.d.	n.d.	No (m)	n.d.	?	Yes-subset
	SVZ/RMS A cells	Yes	n.d.	n.d.	Yes	Yes-subset	Yes-subset
	Increased in SVZ after injury	n.d.	n.d.	n.d.	n.d.	n.d.	Yes
	Ependymal cells	Yes	n.d.	Yes	Yes	? (l)	Yes-subset
	OB						
	Granule	Yes	?	n.d.	No	No	Yes
	neurons						
	Periglomerular	Yes	?	n.d.	Yes	Yes-subset	Yes
	neurons						
	Accessory OB	n.d.	n.d.	n.d.	n.d.	n.d.	Yes
	neurons						
	*Nonneurogenic regions*	Yes (n)	Yes	n.d.	No	Yes (o)	Yes
	Oligodendro- cytes	?	n.d.	n.d.	No	n.d.	Yes
	Astrocytes	?	No	n.d.	n.d.	n.d.	No
	Astrocytes after injury	n.d.	No (p)	n.d.	n.d.	n.d.	No
	Neurons	?	Yes	n.d.	No	n.d.	Yes-weak
	Nonneural tissue	n.d.	n.d.	n.d.	n.d.	n.d.	Yes-hair follicles

Electron microscopy	SVZ B cells	n.d.	n.d.	n.d.	n.d.	n.d.	Yes
	SVZ C cells	n.d.	n.d.	n.d.	n.d.	n.d.	Yes
	SVZ A cells	n.d.	n.d.	n.d.	n.d.	n.d.	Yes
	Adult ependymal cells	n.d.	n.d.	n.d.	n.d.	n.d.	Yes
	Oligodendro- cytes	n.d.	n.d.	n.d.	n.d.	n.d.	Yes

Cell culture	Neurospheres	n.d.	n.d.	Yes (q)	Yes	n.d.	Yes (r)

n.d.: not determined. ?: not clear

(a) Line 3D6—includes mutation of SHP2 tyrosine phosphatase; (b) nestin-tTA mouse crossed with tetracycline regulated CREB used as readout; (c) Okada et al., 1999; (d) shown *in vitro*; (e) nestin expressed in the same general areas as transgene, cell coexpression not determined; (f) tested postnatally; (g) BrdU labeling index not changed, colabelling with transgene not done; (h) embryonically *in vivo* and *in vitro*; (i) parenchymal GFP+ cells visible in Figure 1(g); (j) SVZ but not dentate gyrus; (k) as determined by BrdU retention; (l) S 100b-labelling in Figure 4(a) looks ependymal not astrocytic; (m) determined by lack of BrdU pulse labelling; (n) glial morphology; (o) not labelled with BrdU; (p) not within 6 hrs post-MCAo; (q) embryonically, self-renewal and multipotency shown; (r) postnataly, self-renewal and multipotency shown.

## Abbreviations

3d V: Third ventricle
cc: Corpus callosum
CNPase: 2′,3′-cyclic nucleotide 3′-phosphodiesterase
cpa: Caudatopallial angle
ctx: Cerebral cortex
Dcx: Doublecortin
E: Embryonic age
eGFP: Enhanced green fluorescent protein
epl: External plexiform layer (olfactory bulb)
ft: Fiber tract
ge: Ganglionic eminence
GFAP: Glial fibrillary acidic protein
gl: Glomerular layer (olfactory bulb)
gr: Granule layer (of olfactory bulb)
LGE: Lateral ganglionic eminence
lms: Lateral migratory stream
lv: Lateral ventricle
OB: Olfactory bulb
OPC: Oligodendrocyte progenitor cell
P: Postnatal age
pA: Polyadenylation
pgl: Periglomerular layer (of olfactory bulb)
RMS: Rostral migratory stream
Spt: Septum
Str: Striatum
SVZ: Subventricular zone
TK: Thymidine kinase
VZ: Ventricular zone.
